# An Inactivated Novel Genotype Fowl Adenovirus 4 Protects Chickens against the Hydropericardium Syndrome That Recently Emerged in China

**DOI:** 10.3390/v9080216

**Published:** 2017-08-08

**Authors:** Qing Pan, Yanchao Yang, Yulong Gao, Xiaole Qi, Changjun Liu, Yanping Zhang, Hongyu Cui, Xiaomei Wang

**Affiliations:** 1 State Key Laboratory of Veterinary Biotechnology, Harbin Veterinary Research Institute of Chinese Academy of Agricultural Sciences, Harbin 150001, China; panqing20050101@126.com (Q.P.); yangyanchao533@163.com (Y.Y.); ylg@hvri.ac.cn (Y.G.); qxl@hvri.ac.cn (X.Q.); liucj93711@hvri.ac.cn (C.L.); zhyp_77@hvri.ac.cn (Y.Z.); 2 Jiangsu Co-Innovation Center for Prevention and Control of Important Animal Infectious Disease and Zoonoses, Yangzhou 225009, China

**Keywords:** novel FAdV-4, hypervirulent, hydropericardium syndrome, Th2 response, chickens

## Abstract

Since 2015, China has experienced outbreaks of severe hydropericardium syndrome (HPS), associated with a novel genotype and hypervirulent fowl adenovirus serotype 4 (FAdV-4) infection, with a prevalence in various provinces of the country. This has resulted in huge economic losses in the poultry industry. The novel FAdV-4 showed new genome characters, such as the natural deletion of open reading frame (ORF) 19 and ORF 27 (1966 bp), and high pathogenicity toward chickens. These are coupled with severe hydropericardium, inclusion body hepatitis, and mortality rates ranging from 30% to 90%. Although several inactivated and subunit vaccines against the traditional FAdV-4 have been developed, no commercial vaccine against the emerged disease caused by the novel strain has been available until now. The potential risks of infection with this novel hypervirulent FAdV-4 urgently require an effective vaccine. Thus, an inactivated oil-emulsion FAdV-4 vaccine formulated with the novel genotype virus was developed in this study. The vaccine provided a high level of antibody, preferential T helper 2 (Th2) (interleukin-4 secretion) not Th1 (interferon-γ secretion) response, and full protection against a lethal dose of the novel hypervirulent FAdV-4. Therefore, the novel genotype FAdV-4 vaccine is proposed as an attractive candidate to prevent and reduce the spread of HPS in the poultry industry of China.

## 1. Introduction

Fowl adenoviruses (FAdVs), members of the genus *Aviadenovirus*, are separated into five species (designated FAdV-A to FAdV-E). These are based largely on molecular criteria and restriction enzyme digest patterns or twelve serotypes (designated FAdV-1 to 8a and -8b to 11) based on the results of serum cross-neutralization tests [1]. Hepatitis-hydropericardium syndrome (HHS), inclusion body hepatitis (IBH), and gizzard erosion (GE) in chickens and other birds are always associated with single- or co-infection of FAdVs [2,3].

The first FAdVs infection case was reported in Pakistan in 1987 [4], and subsequent outbreaks have been reported in Germany [5,6], Canada [7], Poland [8], Hungary [9], Chile [10], South America [11], Mexico [12], India [13], Korea [14], Japan [15], and China [16], resulting in significant economic losses to the poultry industry. Almost all the FAdVs could induce IBH with or without mortality, while fowl adenovirus serotype 4 (FAdV-4) plays a primary role in the aetiology of HPS with a mortality that ranges from 10 to 100 percent [12,16]. Since the identification of the causative agent of HPS and IBH, various live and killed vaccines against FAdV-4 have been developed that have provided considerably effective protection against the conventional hypervirulent strain [17,18,19]. Furthermore, several subunit vaccines were also constructed, notably rPenton and rFiber-2, which can provide a 90% and 96.4% protection, respectively [20,21].

Unfortunately, severe HPS induced by a novel hypervirulent FAdV-4, with high mortality rates ranging from 30% to 100% has emerged across several different areas in China since 2015 [22,23]. Given the complete genome sequence analysis, Chinese hypervirulent FAdV-4 strains show many new characteristics, such as the natural large deletions of open-reading frame (ORF)19 and ORF27, and are identified as a novel genotype [24,25,26]. Furthermore, all vaccines previously developed were based on, and used for the conventional strains. There is no commercial vaccine against FAdV-4, especially the emerged HPS caused by the novel genotype strain in China. The potential risks of infection with this novel hypervirulent FAdV-4 urgently require an effective vaccine. Thus, an inactivated novel genotype FAdV-4 vaccine was developed in this study, and the protective efficacy was evaluated by challenging specific-pathogen-free (SPF) chickens with lethal dose of hypervirulent FAdV-4. For the first time, the novel genotype FAdV-4 vaccine is developed and proposed as an attractive candidate to prevent the spread of HPS and reduce the economic losses to the poultry industry of China.

## 2. Materials and Methods

### 2.1. Fowl Adenovirus Serotype 4

FAdV-4 strain HLJFAd15 was isolated from the field layers in 2015 in Heilongjiang Province, China [16], and identified as a novel genotype of FAdV-4 based on the complete genome sequence (GenBank No. KU991797).

### 2.2. Detection of the Titration of FAdV-4

The median tissue culture infective dose (TCID_50_) of HLJFAd15 was determined using 24-well plates according to the methods previously reported [27]. Briefly, primary chicken embryo liver (CEL) cells were prepared from 14-day-old SPF chicken embryos and cultured with M199 medium (Hyclone, Logan, UT, USA), with addition of 10% foetal bovine serum (Ausbian, Shanghai, China). CELs were inoculated with 10-fold dilutions from 10^−3^ to 10^−10^ of virus stocks and incubated at 37.5 °C with 5% CO_2_ for 5 days. Cytopathic effect (CPE) was observed and the TCID_50_ were calculated according to the Reed and Muench method [28]. For median embryo lethal dose (ELD_50_) of HLJFAd15, 9-day-old SPF chicken embryos were infected with serial 10-fold dilutions of HLJDAd15 (0.2 mL) onto the chorioallantoic membranes of each embryo and incubated in a humidified atmosphere (55%) at 37 °C for 10 days. The livers of the embryos died between 3 and 7 days and were harvested with the ELD_50_ being determined using the Reed–Muench method [16,28].

### 2.3. Production of Inactivated FAdV-4 Vaccine

Virus strain HLJFAd15 was propagated on primary chicken embryo liver (CEL) cells and used for vaccine production and challenge strain. For inactivation of the virus, formaldehyde was added to the virus culture medium, harvested from FAdV-4 infected CEL cells with a concentration of 0.2% in the final product [19]. The formaldehyde inactivated antigen solution was emulsified with oil adjuvant at a ratio of 25:75 (*w*/*w*). The immunized dose of the oil-emulsion vaccine was 10^6^ TCID_50_ each time in 0.5 mL per chicken.

### 2.4. Experiment Animals

SPF chickens were obtained and maintained in the Experimental Animal Center of Harbin Veterinary Research Institute (HVRI), China. The animal experiments with chickens were approved by the Ethical and Animal Welfare Committee of Heilongjiang Province, China (License No. SQ20150508).

### 2.5. Immunization and Challenge

Eighty SPF chickens were randomly divided into 4 groups, including 30 birds in the Single IM (immunization) group, 30 in the Double IM group, 10 in the Infection Ctrl. (Control) group, and 10 in the Negative Ctrl. group. Chickens were immunized intramuscularly with 0.5 mL vaccine containing 10^6^ TCID_50_ FAdV-4 antigens per chicken. Birds in the Single IM group were inoculated with the vaccine at 21 days old, while birds in the Double IM group were immunized at 7 and 21 days old, respectively. Birds in the Infection Ctrl. group and Negative Ctrl. group were administered with 0.5 mL phosphate buffer saline (PBS) at the same time points. Two weeks after the last immunization [19], animals were challenged with 10^6^ ELD_50_ of HLJFAd15 in 0.2 mL PBS through oral administration.

### 2.6. Sample Collection

Serum samples were obtained for antibody and cytokine assays at four time points: 7 and 14 days post the first and second immunization. Tissue samples, including the heart, liver, spleen, lung, kidney, thymus, and bursa, were collected 5 days post challenge for histopathology assay and virus quantification [29].

### 2.7. Antibody and Cytokine Assay

FAdV-specific antibodies were tested using a commercial enzyme-linked immunosorbent assay (ELISA) kit according to the manufacturer’s instructions (BioChek, Scarborough, ME, USA). The cytokines (T helper 2 (Th2) cytokine interleukin (IL)-4 and Th1 cytokine interferon (IFN)-γ) [30] in the serum were also checked using commercial ELISA kits (Cloud-Clone, Houston, TX, USA).

### 2.8. Real-Time PCR for Virus Shedding

The total DNA of 0.1 g tissue samples was extracted from tissue homogenates using a DNeasy Tissue kit (Qiagen, Hilden, Germany) according to the manufacturer’s instructions. Real-time PCR was performed with a LightCycler 480 real-time thermocycler (Roche, Rotkreuz, Switzerland). The primers were designed at L1 region of *hexon* as follows: forward primer 5′-CAGTTCATTTCCGCCACC-3′, and reverse primer 5′-GCAGCCGTTGAGCCTTTT-3′. The relative TaqMan probe was a 23 bp oligonucleotide: 5′(FAM)-TCTGTCGTGACATTTCGGGTGGG-3′(TAMRA). The reactions were conducted with a predenaturation step at 95 °C for 5 min, an amplification at a melting temperature of 95 °C for 10 s, and an annealing/elongation at 65 °C for 40 s [29,31]. The fluorescent signal was collected during the elongation step. A 341 bp fragment containing the probe sequence was cloned into a pEASY-T1 vector from 10^1^ to 10^11^ copies/μL, and were used to produce a standard curve. The final concentration was calculated in copy numbers in one mg tissue samples [29].

### 2.9. Histopathology

Tissue samples were fixed in 10% formalin for 48 h at room temperature (RT), and then routinely processed, embedded in paraffin wax, and cut into 5-μm sections. The sections were stained with hematoxylin and eosin (HE), and then examined using light microscopy.

### 2.10. Statistical Analysis

Statistical analyses were performed using the GraphPad Prism package (GraphPad Software, La Jolla, CA, USA). The statistical significance of the difference between the two groups was evaluated by Student’s *t*-test and between more by one-way ANOVA. Differences were considered to be significant at * *p* < 0.05 or ** *p* < 0.01.

## 3. Results

### 3.1. Antibody Responses of Vaccinated Chickens

**Figure 1:**
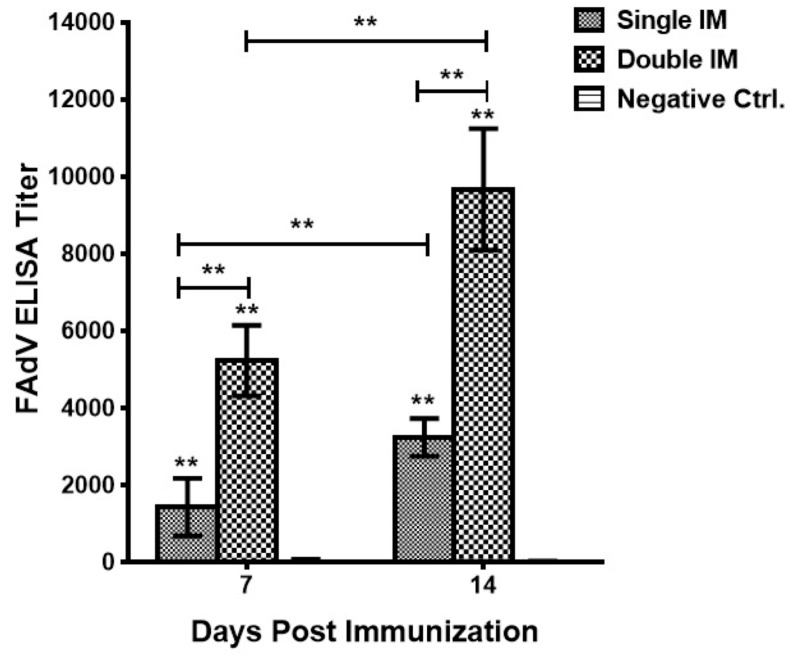
Fowl adenovirus (FAdV)-specific antibody responses induced 7 days and 14 days post single and double immunization. Chickens in the Single immunization (IM) group produced higher antibody response than the Negative Ctrl. group (*p* < 0.01), and the double immunization boosted the immune response more than single immunization (*p* < 0.01). Significant differences between different experimental groups were evaluated at (** *p* < 0.01).

Specific antibody responses elicited after immunizations were measured by titrating the serum of control, and vaccinated chickens against FAdVs by ELISA. In general, chickens in the Negative Ctrl. group showed negative results across the experiment, while chickens in both single and double IM groups were positive and the antibody titers were significantly higher than the Negative Ctrl. group (*p* < 0.01). The results (Figure 1) showed that the magnitude of antibody response was time dependent, and that the antibody titers were significantly higher (*p* < 0.01) at 14 days post immunization (dpi) than 7 dpi, both for single and double IM. Moreover, the second immunization could significantly (*p* < 0.01) boost the antigen-specific antibody responses.

### 3.2. Cytokine Production of SPF Chickens

**Figure 2:**
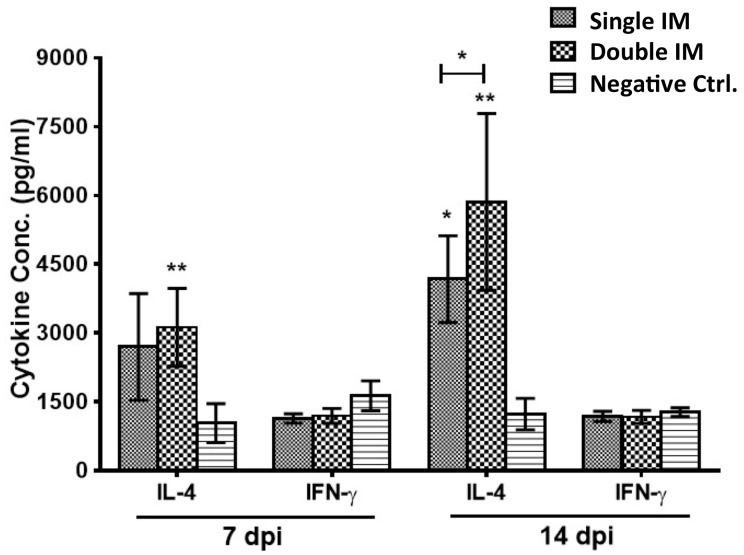
FAdV-4 specific T helper 1 (Th1)/Th2 cytokines responses. Th1 (interferon (IFN)-γ) and Th2 (interleukin (IL)-4) cytokines in serum of chickens were detected by ELISA. A strong Th2 response was induced in the vaccinated chickens. Significant differences between Th1 and Th2 cytokines (IFN-γ and IL-4) were evaluated at (* *p* < 0.05) or (** *p* < 0.01).

Cytokines IL-4 (Th2 response) and IFN-γ (Th1 response) in the serum of chickens were detected 7 and 14 days after the first and second immunization by ELISA. The data (Figure 2) showed that the IL-4 concentrations of chickens in the Single IM (*p* < 0.05) and Double IM (*p* < 0.01) groups were significantly higher than the Negative Ctrl. group at 14 dpi. The IL-4 levels of Double IM groups were significantly higher (0.01 < *p* < 0.05) than the Single IM group at 14 dpi, but there was no difference (*p* > 0.05) between them at 7 dpi. For the IFN-γ assay, there was no difference (*p* > 0.05) between the two immunized groups and the control group at any detected time points.

### 3.3. Protective Efficacy of the Vaccine

**Figure 3:**
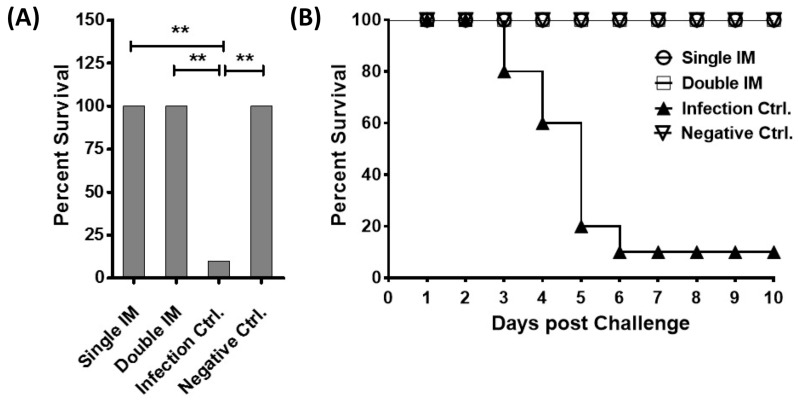
Protective efficacy of the vaccine in challenge experiment. (**A**) Survival rates of chickens in the immunized groups, challenge control group and negative control group; (**B**) The survival curve of the challenge experiment. Significant differences were evaluated at (** *p* < 0.01).

To evaluate the protective efficacy of the inactivated vaccine, the chickens were infected with 10^6^ ELD_50_ of HLJFAd15 in 0.2 mL PBS through oral administration and observed for 10 days. The results showed that the vaccine induced 100% protection (Figure 3A) against the lethal dose of hypervirulent FAdV-4. No SPF chicken died in the two immunized groups (Single IM and Double IM) and the Negative Ctrl. group, whilst 90% of the birds in the Infection Ctrl. group died between 3 to 6 days post infection (Figure 3B). The protective rates of single or double immunization groups were significantly higher (*p* < 0.01) than the infection control group.

### 3.4. Virus Shedding of HLJFAd15 in Different Tissues

**Figure 4:**
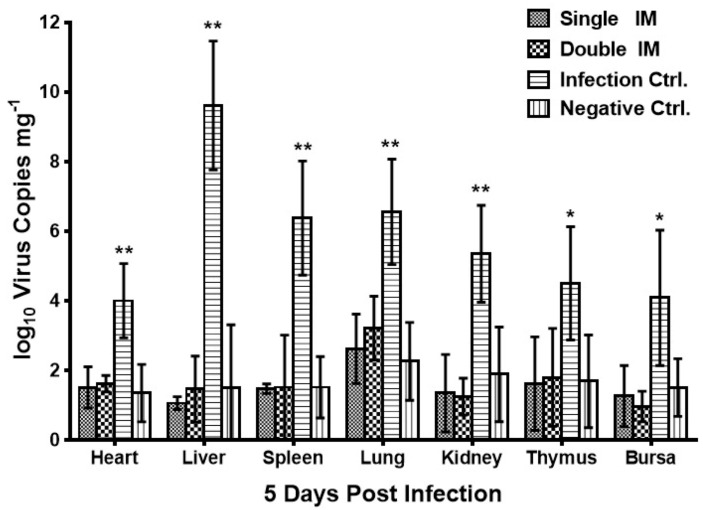
Virus shedding in chickens of different groups at 5 days post infection. Significantly higher levels of virus were detected in different tissues of the chickens in the Infection Ctrl. group. There was no significant difference between chickens in the two immunized groups and the Negative Ctrl. group. Significant differences between different experimental groups were evaluated at (* *p* < 0.05) or (** *p* < 0.01).

The virus loads in four dead birds of the Infection Ctrl. group and five artificial sacrificed birds in other three groups were detected by real-time PCR at five days post infection (Figure 4). For virus concentrations in the heart, liver, spleen, lung, kidney, thymus, and bursa, there was no difference (*p* > 0.05) between both the immunized groups and the negative control group. However, the virus concentrations of birds in the Infection Ctrl. group were significantly higher (*p* < 0.01 for the heart, liver, spleen, lung, and kidney; 0.01 < *p* < 0.05 for the thymus and bursa) than birds in negative Ctrl. group.

### 3.5. Histopathology in Different Tissues

**Figure 5:**
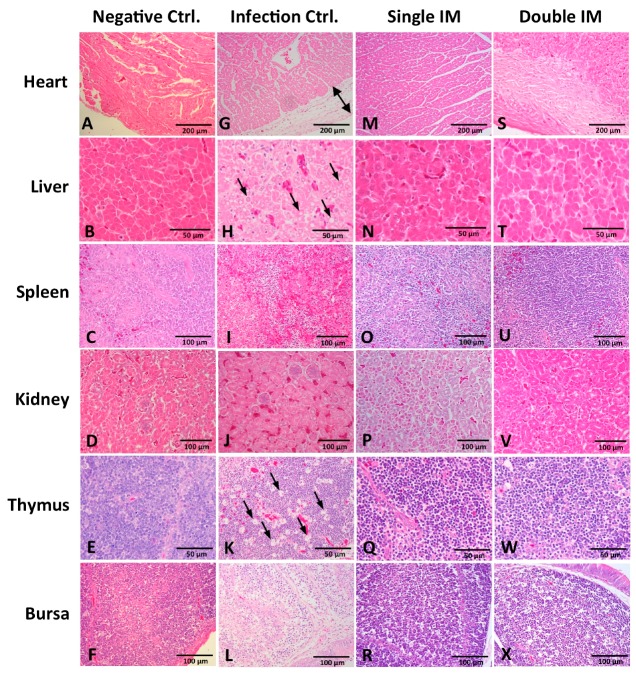
Histopathology in tissues at 5 days post challenge (hematoxylin and eosin (HE) stain). There was no significant histopathology change in tissues of chickens in the Negative Ctrl. group (**A**–**F**), Single IM group (**M**–**R**), and Double IM group (**S**–**X**). Massive pathological damages were observed in chickens of the Infection Ctrl. group: (**G**) severe edema and a small amount of inflammatory cells appeared in the epicardium of the heart. (**H**) Degeneration, vacuolar necrosis and basophilic inclusion bodies presented in liver cells. (**J**) no obvious changes showed in the kidney. (**K**) Large numbers of macrophage proliferations and cortical lymphocyte depletion appeared in the thymus. Severe reduction and necrosis of lymphocytes showed in the spleen (**I**) and bursa (**L**).

Tissues of the heart, liver, spleen, kidney, thymus, and bursa of chickens in different groups were collected five days post challenge and fixed, cut into sections, and stained with HE (Figure 5). For chickens in the Infection Ctrl. group, massive pathological damages were observed in various tissues. Severe edema and small amounts of inflammatory cells appeared in the epicardium of the heart (Figure 5G). Degeneration, vacuolar necrosis, and basophilic inclusion bodies were present in liver cells (Figure 5H). A large number of macrophage proliferations and cortical lymphocyte depletion appeared in the thymus (Figure 5K). Severe reduction and necrosis of lymphocytes showed in the spleen (Figure 5I) and the bursa (Figure 5L). Generally, for the histopathology damage, there was no significant difference between the chickens in the two immunized groups and the Negative Ctrl. group.

## 4. Discussion

As reported, FAdV-4 infection causing HPS and IBH has emerged in 2015 across different provinces in China, inducing huge economic losses for poultry production [16,23]. The FAdV-4 strain was isolated and characterized as a novel genotype [24,26]. Although many studies have been conducted on inactivated or recombinant subunit vaccines consisting of several serotypes of FAdVs against the conventional FAdV-4 strains [32,33,34,35,36,37], no vaccine has been developed and evaluated for the HPS associated with the novel hypervirulent FAdV-4 [26,38]. In this study, an inactivated novel genotype FAdV-4 vaccine formulated with oil-emulsion was developed, and the immune responses and protective efficacy were evaluated by challenging SPF chickens with a lethal dose of the novel hypervirulent FAdV-4. This process was to find an attractive candidate to reduce and control the prevalence of HPS in China.

Because the efficacy of the inactivated vaccine is related to an induction of the humoral immune response, the antibody level of vaccinated chickens could be a useful criterion for the evaluation of inactivated vaccine [19]. The inactivated novel genotype FAdV-4 vaccine developed in this study is capable of inducing high levels of antigen-specific antibody and abundant secretion of IL-4 not IFN-γ, suggesting that a strong Th2 response was introduced [30]. High levels of IL-4 secretion could further stimulate the activation of B cells to enhance the antigen presenting ability, and produce higher antibody titre to neutralize the virus infection. Upon receiving a lethal dose of the novel FAdV-4, full protections were provided in chickens immunized with the vaccine once or twice. Although single immunization is capable of providing a hundred percent protection while saving the vaccine cost and extra work, double immunization was recommended because of the higher antibody responses and secretion of Th2 cytokine IL-4.

In addition, the chickens in the challenge group showed severe HPS and IBH, which were typical clinical signs of novel FAdV-4 infection [16,23]. For vaccinated chickens, no obvious clinical signs and pathological damage were observed in chickens of both the single and double immunization groups, suggesting the safety and sufficient protection of the vaccine. As reported, the novel genotype FAdV-4 has specificities of continuous infection and cross-transmission between chickens and ducks [29]. This suggests that the infected chickens could continually contain and shed the virus to the environment for a long time. Therefore, the control of virus shedding is a core part of reducing the prevalence of HPS. Fortunately, the virus shedding of chickens in the immunized group is equal to chickens in the negative control group, suggesting that the advantage of the vaccine is in the effective control of the horizontal spread of HPS. However, the cross-protection of the vaccine against the conventional FAdV-4 or other serotypes of FAdVs and the further mechanisms of immune responses require further investigation.

## 5. Conclusions

In conclusion, an inactivated novel genotype FAdV-4 vaccine was developed, which elicited high levels of antigen-specific antibodies and Th2 cytokine IL-4, while providing full protection against the HPS. Single immunization was able to provide a hundred percent protection and efficaciously control the horizontal spread of HPS. Although several FAdV-4 vaccines for the conventional strains have been developed, this inactivated vaccine is the first one against the novel genotype strain, and is proposed as an attractive candidate to control the spread of HPS in the poultry industry of China.
